# Cystatin-c May Indicate Subclinical Renal Involvement, While Orosomucoid Is Associated with Fatigue in Patients with Long-COVID Syndrome

**DOI:** 10.3390/jpm13020371

**Published:** 2023-02-19

**Authors:** Laszlo Zavori, Tihamer Molnar, Reka Varnai, Andrea Kanizsai, Lajos Nagy, Bence Vadkerti, Balazs Szirmay, Attila Schwarcz, Peter Csecsei

**Affiliations:** 1 Salisbury NHS Foundation Trust, Salisbury SP2 8BJ, UK; 2 Department of Anaesthesiology and Intensive Care, Medical School, University of Pecs, 7624 Pecs, Hungary; 3 Department of Primary Health Care, Medical School, University of Pecs, 7624 Pecs, Hungary; 4 Department of Dentistry, Medical School, Pecs, University of Pecs, 7624 Pecs, Hungary; 5 Department of Applied Chemistry, University of Debrecen, 4032 Debrecen, Hungary; 6 Department of Laboratory Medicine, Medical School, University of Pecs, 7624 Pecs, Hungary; 7 Department of Neurosurgery, Medical School, University of Pecs, 7624 Pecs, Hungary

**Keywords:** long-COVID syndrome, cystatin-c, orosomucoid, l-arginine, symmetric dimethylarginine, fatigue, renal function

## Abstract

Long-COVID syndrome is associated with high healthcare costs, but its pathophysiology is not yet fully understood. Inflammation, renal impairment or disturbance of the NO system emerge as potential pathogenetic factors. We aimed to investigate the relationship between symptoms of long-COVID syndrome and serum levels of cystatin-c (CYSC), orosomucoid (ORM), l-arginine, symmetric dimethylarginine (SDMA) and asymmetric dimethylarginine (ADMA). A total of 114 patients suffering from long-COVID syndrome were included in this observational cohort study. We found that serum CYSC was independently associated with the anti-spike immunoglobulin (S-Ig) serum level (OR: 5.377, 95% CI: 1.822–12.361; *p* = 0.02), while serum ORM (OR: 9.670 (95% CI: 1.34–9.93; *p* = 0.025) independently predicted fatigue in patients with long-COVID syndrome, both measured at baseline visit. Additionally, the serum CYSC concentrations measured at the baseline visit showed a positive correlation with the serum SDMA levels. The severity of abdominal and muscle pain indicated by patients at the baseline visit showed a negative correlation with the serum level of L-arginine. In summary, serum CYSC may indicate subclinical renal impairment, while serum ORM is associated with fatigue in long-COVID syndrome. The potential role of l-arginine in alleviating pain requires further studies.

## 1. Introduction

The term ‘long COVID’ is used to describe symptoms that continue or develop after an acute SARS-CoV-2 infection including both ongoing symptomatic COVID-19 (from 4 to 12 weeks) and post-COVID-19 syndrome (12 weeks or more) [1]. The symptoms of long COVID include fatigue, dyspnea, cardiac abnormalities, cognitive impairment, sleep disturbances, muscle and joint pain, concentration problems, and headache [2,3]. The pathophysiology of long-COVID syndrome has been intensively researched but not yet fully explored. Since this disease affects several organs with a wide spectrum of manifestations, the direct or indirect involvement of many organ systems can be verified [3]. Assumed mechanisms contributing to the pathogenesis of long-COVID syndrome include viral persistence after initial disease, persistent inflammation and autoimmunity [2]. Involvement of the renal system is well documented in the acute phase of SARS-CoV-2 infection [4,5], and growing evidence suggests a decline in renal function in the 6-to-12-month follow-up period even in patients without apparent signs of renal failure [6]. However, the relationship between kidney function and the number and severity of long-COVID symptoms is still unclear. Serum level of cystatin C (CYSC) is a more precise marker of kidney function than serum creatinine level [7] and has the ability to indicate ‘preclinical’ kidney dysfunction [8]. As CYSC is a proven prognostic factor in acute COVID-19 disease [9,10,11], it seems reasonable to measure it during follow-up in patients with long-COVID syndrome. Orosomucoid (ORM) or alpha-1-acid glycoprotein is an acute-phase protein. It plays a role in limiting the harmful effects of the inflammatory response by reducing tissue damage [12]. As a non-specific inflammatory marker, serum ORM concentration is elevated during infections, malignancies and autoimmune diseases [13]. The L-arginine pathway metabolites (L-arginine, asymmetric [ADMA] and symmetric [SDMA] dymetilarginine) play a pleiotropic role in many biological processes, especially in the control of endothelial and immune activities [14]. Moreover, their elevated levels are predictive of in-hospital mortality in the acute phase of SARS-CoV-2 infection [15]. We hypothesized that subclinical inflammation, renal damage, and dysregulation of the L-arginine pathway may persist for several months resulting in permanent symptoms that manifests as a long-COVID phenotype. To test our assumption, we measured serum CYSC, ORM, L-arginine, ADMA and SDMA levels in patients with long-COVID syndrome. Our primary goal was exploring potential correlations among the long-COVID symptoms and the measured molecules and describing the relationship between these markers and symptom severity. Finally, we looked for correlations between serum anti-spike immunoglobulin (S-Ig) and anti-nucleocapsid immunoglobulin (NC-Ig) levels of patients with long-COVID syndrome and the serum level of CYSC, ORM, L-arginine, ADMA and SDMA, respectively, all measured at the baseline visit.

## 2. Methods

### 2.1. Patients and Protocol

We conducted a prospective observational study at a tertiary reference hospital (University of Pecs). A total of 158 patients with long-COVID syndrome from November 2020 to September 2021 were screened. Each patient was referred by general practitioners (GP) operating in the University area, after preliminary screening. GPs checked the following inclusion criteria: the patient (i) had a confirmed SARS-CoV-2 infection (all participants had at least one positive PCR test or a positive antigen test); (ii) shows symptoms consistent with long-COVID syndrome; and (iii) at the time of recruitment, all participants were presented beyond 30 days after symptom onset and remained symptomatic after the acute phase. Eligible patients presenting with long-COVID symptoms at the GP office were referred to the post-COVID outpatient clinic (baseline visit) for assessment. Exclusion criteria were: (i) preexisting malignant or active autoimmune disease; (ii) immunosuppressive therapy; (iii) acute coronary syndrome; (iv) vaccination against SARS-CoV-2; and (v) known renal dysfunction; (vi) patients ≤ 18 years old; (vii) intensive care unit admission and (viii) any condition that might interfere with the assessment of symptoms. During the baseline visit we repeatedly checked compliance with the inclusion criteria and the patients gave their written informed consent to the study. After routine safety studies including blood pressure, ECG recording, and blood sampling for routine parameters (e.g., white blood cell [WBC], hemoglobin, glucose, creatinine, gamma-glutamyl transferase [GGT], D-dimer, C-reactive protein [CRP], hs-Troponine-T, lactate-dehydrogenase [LDH] levels), patients completed a structured questionnaire that related to the symptoms and their severity. We defined a complaint as a post-COVID symptom if it (i) persisted after the index illness; (ii) appeared after the acute period (30 days after symptom onset); (iii) was not present before the acute infection; (iv) persisted for at least 5 days; and (v) was without other obvious cause behind it.

The symptoms in the survey were systematically grouped, such as general (fatigue or easy fatigability, fever), respiratory (dyspnea, cough), cardiovascular (palpitation, chest pain), neuropsychiatric (headache, sleep disturbance, memory deficit, brain fog, depression and irritability, loss of smell), dermatologic (hair loss and rash), gastrointestinal (abdominal pain, nausea, diarrhea), and musculoskeletal system (myalgia and joint pain). To evaluate the level of impact by the symptoms, participants were asked to score each symptom from 0 (have no problem) to 10 (have extreme problem) on a visual analogue scale (VAS). Laboratory testing and measurement of antibody levels were performed by the Department of Laboratory Medicine blinded for patients’ data. Demographic and index disease related data were collected from the participants. Further information was obtained from patient’s electronic medical records, including the relevant clinical data such as the onset time of the first symptoms, details of hospitalization, in- and outpatient care, antiviral medication (favipiravir or remdesivir), need for oxygen supplementation, smoking habits, and BMI. 

### 2.2. Blood Sampling and Assay

Serum blood samples were drawn into Vacutainer^®^-tubes from patients at the baseline visit to determine concentrations of ORM, CYSC, l-arginine, ADMA and SDMA. The samples were centrifuged within 10 min at 3500 rpm for 15 min. The supernatant was immediately stored in aliquot at −80 °C until further processing. L-arginine, ADMA and SDMA were measured in the plasma by high-performance liquid chromatography after derivatization in collaboration with the Department of Applied Chemistry at the University of Debrecen, Hungary. Serum ORM and CYSC were measured by automated immune turbidimetric method using commercially available kits (Tina-quant α1-Acid Glycoprotein Gen.2, Roche Diagnostics GmbH (Mannheim, Germany) and Cystatin C FS kit, DiaSys Diagnostic Systems GmbH (Holzheim, Germany), respectively) at Department of Laboratory, University of Pecs, Hungary. Antibodies against SARS-CoV-2 were assessed in the peripheral blood at baseline visit by a fully-automated cobas e801 analyzer (Roche Diagnostics, Switzerland). We used an age-matched healthy population that had not been infected with COVID as a control group.

### 2.3. Ethical Considerations

The study was approved by the Hungarian Medical Research Council (IV/2505-3/2021/EKU). All procedures were performed in accordance with the ethical guidelines of the 1975 Declaration of Helsinki. All participants provided written informed consent before enrollment in the study.

### 2.4. Statistical Analysis

Data were evaluated using SPSS (version 11.5; IBM, Armonk, NY, USA). The Kolmogorov–Smirnov test was applied to check for normality. To analyze demographic and clinical factors, the chi-square test was used for categorical data while the Student *t* test was applied to quantitative values. Non-normally distributed data were presented as median and interquartile range (25th–75th percentiles) and were compared with the use of Mann–Whitney test. Correlation analysis was performed by calculating Spearman’s correlation coefficient (rho). A *p*-value < 0.05 was considered statistically significant.

## 3. Results

Of the 158 patients who underwent screening, 114 were finally included in the study and 44 patients were excluded because of autoimmune/malignant disease (5), vaccination against SARS-CoV-2 (4), ongoing immunosuppressant therapy (7), chronic renal dysfunction (5), withdrawal of consent during study process (4), deviation in the routine safety laboratory test (8), unavailable biomarker measurements (8), asymptomatic on baseline visit (3). The median serum ORM (g/L) level was significantly different from the median serum level of the age-matched healthy control group (study group: 0.92, IQR: [0.76–1.07] vs. healthy control: 0.7, [0.62–0.82], *p* = 0.023). Serum cystatin C (mg/L) values in the patient group and in the control group: 0.95, [0.84–1.08] vs. 0.86, [0.80–0.94], *p* > 0.05.

The mean (SD) age was 50.5 (±12) years and 72 (63.2%) of the patients were female. Forty-one (36%) patients had a history of hospitalization related to index SARS-CoV-2 infection and the median number of post-COVID symptoms was five (interquartile range, IQR: 3–8). An overview of baseline characteristics in clusters of patients grouped based on serum ORM and CYSC median value are presented in Table 1. 

The occurrence of fatigue and dyspnea in the group with higher ORM concentration is significantly more common than in the group with concentration below the median. CRP and NLR were higher in the group with ORM concentration above the median, while troponin and D-dimer were higher in the group with CYSC level above the median. The details of the correlations among baseline visit laboratory data, long COVID symptoms and serum ORM and CYSC levels are shown in Table 2.

The serum creatinine and CYSC concentrations measured at the baseline visit showed a strong positive correlation with the serum SDMA levels. On the other hand, the severity of abdominal and muscle pain indicated by patients at the baseline visit showed a negative correlation with the serum level of L-arginine. Cross-sectional analysis among L-arginine, ADMA and SDMA measured on baseline visit and clinical variables are shown in Table 3.

A binary logistic regression model was used to predict the appearance of fatigue among patients with long-COVID syndrome at the baseline visit, including several variables such as age, serum ORM, sex and hospitalization during index SARS-CoV-2 infection. Serum ORM measured at baseline visit with an OR of 9.670 (95% CI: 1.34–9.93; *p* = 0.025) independently predicted fatigue in patients with long-COVID syndrome (Table 4). 

In univariate analysis, serum CYSC concentration positively correlated with the S-IG concentration measured at the baseline visit. In order to determine whether serum CYSC is independently associated with serum S-Ig level, we performed a binary logistic regression analysis involving all parameters. This demonstrated a significant correlation to serum S-Ig level and revealed that serum CYSC was independently associated with the level of serum S-Ig measured at the baseline visit (OR: 5.377, 95% CI: 1.822–12.361; *p* = 0.02) (Table 5).

Significantly higher liver function values and CYSC levels were observed in those receiving antiviral treatment during index disease, but no differences were observed in other kidney function parameters between the two groups (Table 6).

## 4. Discussion

The present study provided the following main results: (i) serum level of CYSC is an independent predictor of serum SARS-CoV-2-S-Ig level and indicates subclinical kidney damage associated with a more severe initial infection; (ii) the serum CYSC level shows a close positive correlation with the concentration of SDMA; (iii) serum ORM level is an independent predictor of the occurrence of fatigue reported by long-COVID patients and shows association with fatigue severity; and finally (iv) lower serum L-arginine levels showed an inverse correlation with the severity of abdominal and muscle pain in patients with long-COVID syndrome. 

Significantly higher serum S-Ig levels were observed both in patients requiring hospitalization during the acute infection and in those presented with higher CYSC levels at the baseline visit. It was shown that patients with higher serum CYSC level have an increased risk for severe COVID-19 disease and higher mortality [9,10,16]. Furthermore, patients who survived COVID-19 exhibited an increased risk of kidney-related outcomes in the post-acute phase of the disease [17]. There is evidence that acute kidney injury in the acute phase of COVID-19 was closely related to the longitudinal decline of renal function up to nearly 1 year after the onset of symptoms [18]. In this study, creatinine-based estimated glomerular filtration rate (eGFR) was used, but it is known that CYSC is a much more sensitive marker of kidney function than serum creatinine level [19,20]. Moreover, elevated CYSC, which occurs earlier than serum creatinine, is helpful in the early detection of kidney dysfunction and may have a better predictive value for disease severity in COVID-19 patients [11]. In one of our previous studies, we found a significantly higher anti-S-Ig titer in long-COVID patients requiring hospitalization, while the level of anti-NC-Ig showed no correlation with hospitalization [21]. Our results were confirmed by other studies [22,23]. Based on these findings, it is likely that the renal function deviation in patients with long-COVID disease indicated by CYSC above the median and without a simultaneous increase in creatinine is the result of a more serious initial COVID-19 episode requiring hospitalization. The more severe SARS-CoV-2 infection in the acute phase may be responsible for the impairment of kidney function indicated by CYSC at the subclinical level in our study, which in contrast does not show a correlation with the strength of long-COVID symptoms. SARS-CoV-2 spike Ig levels are higher in hospitalized patients with more severe initial infection, while CYSC levels in long-term COVID patients indicate renal effects of the same more severe condition during follow-up. In this way, the serum CYSC level is an indirect sign of the previously suffered subclinical kidney damage not accompanied by increased creatinine in patients with long COVID syndrome. In our study, SDMA showed a strong positive correlation with the serum level of CYSC, but at the same time, it shows a lower degree of correlation with the frequency of hospitalization compared to CYSC. Based on a meta-analysis, SDMA is an excellent and sensitive marker of kidney function [24]. Among the three parameters measured in our study (creatinine, CYSC, SDMA), CYSC seems to be the most sensitive in terms of kidney function [25,26]. While the serum level of CYSC is an independent predictor of the S-Ig level measured at the baseline visit, no correlation was observed between serum SDMA and SARS-CoV-2 spike antibody levels. Based on these observations, we can assume that the subclinical kidney damage indicated by an increased serum CYSC level is different from the kind of kidney damage highlighted by the serum SDMA in our cohort of long-COVID patients. Since the CYSC and ORM levels were also significantly higher in the group of patients taking antiviral medication, it was suggested that the potential side effects of such medications could manifest in either impaired kidney function or other unknown organ dysfunctions. Interestingly, neither the creatinine nor the SDMA levels differed in this comparison; thus, it is likely that the increase in the level of CYSC and ORM are not associated with the drugs administered during the index disease. 

The serum ORM level measured at the baseline visit proved to be an independent predictor of the occurrence of fatigue and showed a close correlation with its severity in our cohort.

ORM was identified as a predictor of the 5-year risk of death from any cause [27]. In a previous study, significantly elevated ORM levels were found in the serum of chronic fatigue syndrome (CFS) patients compared to healthy volunteers [28]. Moreover, serum cortisol levels were found to be modestly reduced in these CFS patients. This result is also consistent with previous studies reporting mild hypocortisolism in CFS patients with hypothalamic–pituitary–adrenal (HPA) axis dysfunction [29].

In another study, serum ORM was found to be an independent predictor of severe exercise-induced fatigue (EIF) [30]. Moreover, in rodent fatigue models fatigue seems to upregulate the level of ORM, which in turn functions as an anti-fatigue protein to enhance muscle endurance [31]. Glucocorticoids are known as an important regulator of ORM expression [32]; however, short-term administration of prednisolone in healthy volunteers had little effect on ORM levels [33]. The serum steroid levels were also significantly increased in fatigued rodents, indicating that it may be responsible for the increase of ORM [31]. Recent evidence suggests that a short course of corticosteroids reverted immune alterations and improved post-COVID syndrome [34], and the use of systemic steroids helps facilitate the recovery of long-COVID patients [35]. Steroid therapy seems to be useful in post-COVID conditions affecting the respiratory system as well [36,37]. There is also evidence that adrenal insufficiency might develop several weeks after an acute phase of COVID-19 [38]. CFS is very common in patients with post-COVID syndrome [39] and hypocortisolism has previously been known to be associated with CFS [40]. Considering the abovementioned evidence, it can be assumed that a certain degree of dysfunction of the glucocorticoid system is likely in patients suffering from long-COVID fatigue, which may be indicated by higher ORM levels. Unfortunately, we did not examine glucocorticoid levels in our study, so we cannot confirm the assumption with certainty. At the same time, the increase in the ORM level observed in CFS and the elevated ORM level found in our study in patients with fatigue may suggest a common pathophysiological process behind the two syndromes. 

An inverse correlation was observed between the level of L-arginine and the intensity of abdominal and muscle pain observed in our patient cohort. There is evidence for the analgesic effect of L-arginine. The analgesic effect of L-arginine in patients with persistent pain has long been known [41] and L-arginine supplementation can prevent allodynia and hyperalgesia in painful diabetic neuropathic rats [42] and the nociceptive effect of L-arginine could be pronounced and persistent in rats [43]. L-arginine can improve the clinical symptoms of intermittent claudication in patients with peripheral arterial occlusive disease [44]. The background of the observed correlation between the pain level of long COVID patients and L-arginine level is not yet clear. L-arginine is a common precursor of NO and is displaying both pronociceptive and antinociceptive effects [45], while it can also affect NO levels, which may modulate spinal pain processing [42]. Another potential mechanism is that L-arginine activates the biosynthesis and release of kyotorphin followed by the increased release of met-enkephalin in the brainstem and spinal cord, which refers to its interaction with the opioid system [41]. In addition to the molecules discussed in our study, miRNAs can play an important role in the vascular pathogenetic processes of the long-COVID syndrome, a subsequent investigation of this can be recommended [46]. L-arginine was also proven to increase collagen synthesis in animal models [47]. Increasing or restoring the collagen content in the extracellular matrix (ECM) of injured tissues might contribute to reducing inflammation [48] and consequently might also affect fatigue and pain perception. It would be interesting to examine the effect of vaccination on symptoms or on the serum level of metabolites as there is evidence that the long COVID syndrome can be influenced by anti-COVID vaccines [49]. Similarly, it would be useful to perform serum cystatin c and ORM measurements in the future according to risk factors in a wider population [50].

In conclusion, we found that the serum CYSC level in long-COVID patients is an independent predictor of the SARS-CoV-2 spike IgG level measured at the baseline visit. This is probably an indirect consequence of the subclinical renal involvement caused by a more severe disease since a more severe index SARS-CoV-2-infection resulted in higher spike Ig levels in the follow-up studies. The serum ORM level proved to be an independent predictor of the fatigue observed in long COVID patients, and it also showed a close correlation with its severity. This result makes it likely that fatigue observed in long-COVID syndrome may have a similar pathophysiology to chronic fatigue syndrome. Finally, we found that L-arginine levels in patients with long-COVID syndrome were inversely correlated with lower abdominal and muscle pain severity.

## 5. Limitations 

Our study design has several limitations to acknowledge. First, this study only included patients from a single center, which may have led to sampling bias and limits the generalizability of our findings. Multi-center research is required with higher patient numbers to confirm our results. Secondly, because of the strict exclusion criteria, we only included 114 patients, which may have limited the power of our study. For that reason, our results should be interpreted with caution and should be further corroborated with larger-scale studies. Thirdly, we did not examine our patients’ glucocorticoid levels, making interpreting our results regarding the ORM levels observed less straightforward. Another potential confounding factor is that we did not differentiate between COVID variants, and it is possible that different variants contribute to long-COVID symptoms differently.

## Figures and Tables

**Table 1:** Demographics and acute SARS-COV-2-infection-related variables in patients with higher and lower than the median serum cystatin-c and orosomucoid concentrations measured in long-COVID patients.

		Serum Cystatin-c ^†^ (mg/L)		*p*-Value	Serum ORM (g/L) ^†^		*p*-Value
		Median>, *n* = 59	Median<, *n* = 55		Median>, *n* = 55	Median<, *n* = 59	
Age	years (mean ± SD)	45 ± 11	55 ± 11	*<0.001*	50 ± 13	51 ± 10	0.925
Female	N (%)	39 (66%)	31 (56%)	0.338	34 (62%)	38 (64%)	0.777
Hypertension	N (%)	15 (25%)	20 (36%)	0.211	15 (27%)	22 (37%)	0.407
NIDDM	N (%)	7 (12%)	9 (16%)	0.429	9 (16%)	7 (12%)	0.370
Smoking	N (%)	3 (6%)	1 (2%)	0.620	1 (2%)	3 (5%)	0.376
BMI	mean ± SD	26 ± 5	29 ± 7	0.06	26 ± 7	29 ± 5	*0.007*
Hospitalization	N (%)	13 (22%)	29 (53%)	*0.001*	15 (27%)	27 (44%)	0.063
O_2_-supplementation	N (%)	8 (14%)	16 (29%)	0.065	6 (11%)	17 (29%)	*0.02*
Antiviral medication	N (%)	9 (15%)	30 (55%)	*<0.001*	12 (22%)	27 (46%)	*0.01*
CTss ^‡^	median (IQR)	7 (0–12)	9 (5–13)	0.201	8 (0–12)	9 (5–12)	0.322
Length of hospitalization	(day, mean ± SD)	2 ± 3	4 ± 5	*<0.001*	2 ± 3	4 ± 5	*0.033*
Total number of symptoms related to index infection	median (IQR)	6 (4–7)	5 (4–7)	0.522	5 (3–7)	6 (4–8)	0.401
Duration of smell loss during index disease	(day, mean ± SD)	17 ± 22	9 ± 10	*0.05*	8 (3–21)	4 (0–14)	0.055
IL-6 ^‡^	pg/mL, median (IQR)	30 (17–31)	28 (20–46)	0.820	31 (19–36)	28 (19–42)	0.871
Ferritin ^‡^	µg/L, median (IQR)	469 (302–658)	611 (462–984)	0.391	566 (354–1366)	615 (422–915)	0.792
acute hs-Troponin-T ^‡^	ng/L, median (IQR)	7.6 (5–9)	8 (5–10)	0.998	9 (7–11)	5 (4–9)	0.196
eGFR	median (IQR)	105 (99–114)	94 (84–107)	*<0.001*	101 (86–110)	102 (90–109)	0.636

Abbreviations: N, number; NIDDM, non-insulin-dependent diabetes mellitus; BMI, body mass index; IL-6, interleukin-6; SD, standard deviation; IQR, interquartile range; ORM, orosomucoid; NLR, neutrophil-to-lymphocyte ratio; SARS-CoV-2, severe acute respiratory syndrome coronavirus-2; CTss, computer tomography severity score; hs-Troponin-T, high sensitive Troponin-T. Variables are expressed as mean (SD) or median with interquartile ranges (IQR) when continuous and counts (percentage) when categorical. Significant *p*-values are highlighted in italics; †, measured on follow-up visit; ‡, measured on admission related to index SARS-CoV-2 infection.

**Table 2:** Associations of median serum cystatin-c and orosomucoid concentrations measured in long-COVID patients with variables measured and collected on follow-up visit.

		Serum Cystatin-c ^†^ (mg/L)		*p*-Value	Serum ORM (g/L) ^†^		*p*-Value
		Median>, *n* = 59	Median<, *n* = 55		Median>, *n* = 55	Median<, *n* = 59	
Anti-SARS-CoV-2 S-Ig	U/mL, median, IQR	72 (28–147)	180 (116–482)	*<0.001*	108 (31–279)	143 (65–352)	0.098
Anti-SARS-CoV-2 NC-Ig	U/mL, median, IQR	54 (19–104)	59 (23–115)	0.309	58 (23–107)	54 (24–112)	0.710
Total value of post-COVID symptoms on a VAS scale *	median, IQR	16 (10–19)	15 (10–20)	0.989	15 (6–20)	16 (12–19)	0.338
Number of post COVID symptoms (median, IQR)	median, IQR	6 (3–9)	4 (2–8)	0.200	4 (2–8)	5 (3–8)	0.293
CRP	mg/L, median, IQR	1 (0.5–3)	2 (1–4)	0.052	0.9 (0.5–1.7)	3.3 (1.5–7)	*<0.001*
hsTroponin-T	ng/L, median, IQR	4 (3–5)	5.4 (4–9)	*<0.001*	4 (3–6)	4 (3–8)	0.288
NLR	median, IQR	2 (1.6–2.6)	2.2 (1.8–3.2)	*0.025*	1.9 (1.5–2.6)	2.1 (1.8–3.2)	*0.036*
WBC	G/L, median, IQR	6.1 (4.9–7.3)	6.6 (5.2–8)	0.162	6.1 (4.7–7.8)	6.6 (5.3–7.9)	0.131
Creatinine	µmol/L, median, IQR	66 (57–75)	72 (55–84)	0.081	70 (62–77)	67 (54–78)	0.234
GGT	U/L, median, IQR	17 (14–34)	33 (18–56)	*0.002*	17 (14–30)	33 (18–60)	*<0.001*
D-dimer	µg/L, median, IQR	281 (202–431)	374 (221–546)	*0.028*	352 (221–486)	310 (202–459)	0.606
LDH	U/L, median, IQR	328 (281–357)	355 (308–429)	*0.006*	317 (282–357)	355 (317–411)	*0.001*
Fatigue	N, %	40 (67.8%)	38 (69.1%)	0.882	29 (53%)	48 (81%)	*0.001*
Depression	N, %	9 (15.3%)	6 (10.9%)	0.585	9 (16.4%)	6 (10.2%)	0.410
Dyspnea	N, %	14 (23.7%)	13 (23.6%)	0.991	8 (15%)	19 (32%)	*0.03*
Cardiovascular	N, %	37 (62.7%)	28 (50.9%)	0.257	33 (60%)	31 (52.5%)	0.455
Neurological	N, %	28 (47.5%)	20 (36.4%)	0.259	23 (41.8%)	25 (42.4%)	0.952
Pain	N, %	19 (32.2%)	18 (32.7%)	0.952	15 (27.3%)	22 (37.3%)	0.318
Gastrointestinal	N, %	5 (8.5%)	7 (12.7%)	0.549	6 (10.9%)	7 (11.9%)	0.873
Smell loss	N, %	19 (32.2%)	5 (9.1%)	*0.004*	13 (26.6)	12 (20.3%)	0.821
Fatigue VAS	median, IQR	0 (0–7)	3.5 (0–7)	0.513	0 (0–5)	7 (0–8)	*0.006*

Abbreviations: N, number; SARS-CoV-2, severe acute respiratory syndrome coronavirus-2; VAS, visual analogue scale; S-Ig, spike immunoglobulin; CRP, C-reactive protein; NLR, neutrophil–lymphocyte ratio; GGT, gamma-glutamyl transferase; LDH, lactate dehydrogenase; WBC, white blood cell count. Variables are expressed as mean (SD) or median (IQR) when continuous and counts (percentage) when categorical. Significant *p*-values are highlighted in italics; †, measured on follow-up visit; *, the sum of the VAS score is given for the three most severe symptoms.

**Table 3:** Cross-sectional analysis among L-arginine, ADMA, and SDMA measured on baseline visit and clinical variables.

	L-Arginine ^c^	ADMA ^c^	SDMA ^c^
Hospitalization	0.038	0.161	0.179 *
Length of hospitalization	−0.002	0.119	0.187 *
Age	−0.065	0.100	0.157
Sex	−0.092	−0.159	0.039
Serum Cystatin-C ^a^	−0.039	0.203 *	0.350 **
Serum ORM ^a^	0.031	0.029	−0.030
Serum S-Ig ^a^	0.038	−0.044	0.079
Serum NC-Ig ^a^	−0.094	−0.098	−0.119
hsTroponine-T acute ^b^	−0.410 *	−0.516 **	−0.097
WBC ^a^	−0.028	−0.070	−0.179 *
Creatinine ^a^	0.108	−0.104	0.272 **
GOT ^a^	0.075	0.155	0.184 *
hs-Troponine-T ^a^	0.009	0.026	0.192 *
Abdominal pain VAS	−0.264 *	0.055	−0.020
Joint pain VAS	−0.006	−0.104	−0.235 *
Muscle pain VAS	−0.225 *	−0.060	−0.012
Fatigue VAS	−0.053	−0.039	−0.102
Fatigue	0.044	0.028	−0.030

Abbreviations: ORM, orosomucoid; WBC, white blood cell count; GOT, glutamate-oxaloacetate transaminase; VAS, visual analogue scale; S-Ig, SARS-CoV-2 spike immunoglobulin G; SARS-CoV-2 nucleocapsid immunoglobulin; ^a^ measured on follow-up visit; ^b^ measured on admission related to index SARS-CoV-2 infection; ^c^ µmol/L values are Spearman correlation coefficients (rho). * *p* < 0.05, ** *p* < 0.01.

**Table 4:** Binary logistic regression analysis for variables independently associated with fatigue on follow-up visit in patients with long-COVID syndrome.

Variables	B	Odds Ratio	95% CI	*p*-Value
Age	−0.011	0.989	0.95–1.03	0.564
Serum ORM ^†^	2.269	9.670	1.34–9.93	0.025
Sex	0.792	2.209	0.95–1.03	0.067
Hospitalization	0.324	1.383	0.54–3.56	0.501

ORM, orosomucoid; †, measured on baseline visit.

**Table 5:** Binary logistic regression analysis assessing correlations between anti-SARS-CoV-2 spike antibody (S-Ig) level and demographic/clinical variables. In this model we assigned a binary dependent variable to serum S-Ig level based on the median value of the sample (0: ≤median, 1: >median).

Variables	B	Odds Ratio	95% CI	*p*-Value
BMI	0.068	2.581	0.985–1.164	0.108
Serum ORM ^†^	0.913	0.782	0.239–18.855	0.376
Sex	0.167	0.119	0.456–3.063	0.730
Hospitalization	−1.453	8.169	0.086–0.634	0.004
Serum CYSC ^†^	3.875	5.377	1.822–12.361	0.02
Age	0.004	0.026	0.957–1.053	0.782

ORM, orosomucoid; CYSC, cystatin-C; BMI, body mass index; †, measured on baseline visit.

**Table 6:** Associations between serum renal and liver markers measured on baseline visit and antiviral medication.

	Favipiravir (*n* = 29) vs. No Antiviral Medication	Remdesivir (*n* = 10) vs. No Antiviral Medication
Serum Cystatin-C ^a^	0.001	0.034
Serum Creatinine ^a^	0.823	0.521
Serum SDMA ^a^	0.085	0.844
Serum BUN ^a^	0.476	0.704
Serum GOT ^a^	0.015	0.671
Serum GPT ^a^	<0.001	0.074
Serum GGT ^a^	0.006	0.059

Abbreviations: GOT, glutamate-oxaloacetate transaminase; GPT, glutamate pyruvate alanine aminotransferase; GGT, gamma-glutamyl transferase; BUN, blood urea nitrogen; SDMA, symmetric dimethylarginine; ^a^ measured at baseline visit. The comparisons between groups were performed with the Mann–Whitney U test; *p*-values are reported as exact numbers.

## Data Availability

All relevant data are within the manuscript.

## References

[1] National Institute for Health and Care Excellence (2020). COVID-19 Rapid Guideline: Managing the Long-Term Effects of COVID-19. NICE Guideline. https://www.nice.org.uk/guidance/ng188.

[2] Mehandru S., Merad M. (2022). Pathological sequelae of long-haul COVID. Nat. Immunol..

[3] Nalbandian A., Sehgal K., Gupta A., Madhavan M.V., McGroder C., Stevens J.S., Cook J.R., Nordvig A.S., Shalev D., Sehrawat T.S. (2021). Post-acute COVID-19 syndrome. Naturemedicine.

[4] Migliaccio M.G., Di Mauro M., Ricciolino R., Spiniello G., Carfora V., Verde N., Mottola F.F., Coppola N., Vanvitelli COVID-19 Group (2021). Renal Involvement in COVID-19: A Review of the Literature. Infect. Drug Resist..

[5] Legrand M., Bell S., Forni L., Joannidis M., Koyner J.L., Liu K., Cantaluppi V. (2021). Pathophysiology of COVID-19-associated acute kidney injury. Nat. Rev. Nephrol..

[6] Copur S., Berkkan M., Basile C., Tuttle K., Kanbay M. (2022). Post-acute COVID-19 syndrome and kidney diseases: What do we know?. J. Nephrol..

[7] Dharnidharka V.R., Kwon C., Stevens G. (2002). Serum cystatin C is superior to serum creatinine as a marker of kidney function: A meta-analysis. Am. J. Kidney Dis..

[8] Shlipak M.G., Katz R., Sarnak M.J., Fried L.F., Newman A.B., Stehman-Breen C., Seliger S.L., Kestenbaum B., Psaty B., Tracy R.P. (2006). Cystatin C and prognosis for cardiovascular and kidney outcomes in elderly persons without chronic kidney disease. Ann. Intern. Med..

[9] Zinellu A., Mangoni A.A., Cystatin C. (2022). COVID-19 severity and mortality: A systematic review and meta-analysis. J. Nephrol..

[10] Lin L., Chen X., Chen J., Pan X., Xia P., Lin H., Du H. (2021). The predictive value of serum level of cystatin C for COVID-19 severity. Sci. Rep..

[11] Chen S., Li J., Liu Z., Chen D., Zhou L., Hu D., Li M., Long W., Huang Y., Huang J. (2021). Comparing the Value of Cystatin C and Serum Creatinine for Evaluating the Renal Function and Predicting the Prognosis of COVID-19 Patients. Front. Pharmacol..

[12] Lögdberg L., Wester L. (2000). Immunocalins: A lipocalin subfamily that modulates immune and inflammatory responses. Biochim. Biophys. Acta.

[13] Ceciliani F., Pocacqua V. (2007). The acute phase protein alpha1-acid glycoprotein: A model for altered glycosylation during diseases. Curr. Protein Pept. Sci..

[14] Adebayo A., Varzideh F., Wilson S., Gambardella J., Eacobacci M., Jankauskas S.S., Donkor K., Kansakar U., Trimarco V., Mone P. (2021). l-Arginine and COVID-19: An Update. Nutrients.

[15] Hannemann J., Balfanz P., Schwedhelm E., Hartmann B., Ule J., Müller-Wieland D., Dahl E., Dreher M., Marx N., Böger R. (2021). Elevated serum SDMA and ADMA at hospital admission predict in-hospital mortality of COVID-19 patients. Sci. Rep..

[16] Kleeman S.O., Cordioli M., Timmers P., Khan A., Tober-Lau P., Kurth F., Demichev V., Meyer H.V., Wilson J.F., Ralser M. (2022). Cystatin C is associated with adverse COVID-19 outcomes in diverse populations. iScience.

[17] Bowe B., Xie Y., Xu E., Al-Aly Z. (2021). Kidney Outcomes in Long COVID. J. Am. Soc. Nephrol..

[18] Gu X., Huang L., Cui D., Wang Y., Wang Y., Xu J., Shang L., Fan G., Cao B. (2022). Association of acute kidney injury with 1-year outcome of kidney function in hospital survivors with COVID-19: A cohortstudy. EBioMedicine.

[19] Mussap M., Vestra M.D., Fioretto P., Saller A., Varagnolo M., Nosadini R., Plebani M. (2002). Cystatin C is a more sensitive marker than creatinine for the estimation of GFR in type 2 diabetic patients. Kidney Int..

[20] Helmersson-Karlqvist J., Lipcsey M., Ärnlöv J., Bell M., Ravn B., Dardashti A., Larsson A. (2021). Cystatin C predicts long term mortality better than creatinine in a nationwide study of intensive care patients. Sci. Rep..

[21] Molnar T., Varnai R., Schranz D., Zavori L., Peterfi Z., Sipos D., Tőkés-Füzesi M., Illes Z., Buki A., Csecsei P. (2021). Severe Fatigue and Memory Impairment Are Associated with Lower Serum Level of Anti-SARS-CoV-2 Antibodies in Patients with Post-COVID Symptoms. J. Clin. Med..

[22] Terpos E., Politou M., Sergentanis T.N., Mentis A., Rosati M., Stellas D., Bear J., Hu X., Felber B.K., Pappa V. (2020). Anti-SARS-CoV-2 Antibody Responses in Convalescent Plasma Donors Are Increased in Hospitalized Patients; Subanalyses of a Phase 2 Clinical Study. Microorganisms.

[23] Marklund E., Leach S., Axelsson H., Nyström K., Norder H., Bemark M., Angeletti D., Lundgren A., Nilsson S., Andersson L.M. (2020). Serum-IgG responses to SARS-CoV-2 after mild and severe COVID-19 infection and analysis of IgG non-responders. PLoS ONE.

[24] Kielstein J.T., Salpeter S.R., Bode-Boeger S.M., Cooke J.P., Fliser D. (2006). Symmetric dimethylarginine (SDMA) as endogenous marker of renal function—A meta-analysis. Nephrol. Dial. Transplant..

[25] El-Khoury J.M., Bunch D.R., Hu B., Payto D., Reineks E.Z., Wang S. (2016). Comparison of symmetric dimethylarginine with creatinine, cystatin C and their eGFR equations as markers of kidney function. Clin. Biochem..

[26] El-Khoury J.M., Bunch D.R., Payto D., Reineks E., Wang S. Performance of Symmetric Dimethylarginine Against Other Markers of Kidney Function. https://www.msacl.org/2015_US_Long_Abstracts/201411071218_37806.pdf.

[27] Fischer K., Kettunen J., Würtz P., Haller T., Havulinna A.S., Kangas A.J., Soininen P., Esko T., Tammesoo M.L., Mägi R. (2014). Biomarker profiling by nuclear magnetic resonance spectroscopy for the prediction of all-cause mortality: An observational study of 17,345 persons. PLoS Med..

[28] Sun Y., Zhang Z.X., Liu X. (2016). Orosomucoid (ORM) as a Potential Biomarker for the Diagnosis of Chronic Fatigue Syndrome (CFS). CNS Neurosci. Ther..

[29] Klimas N.G., Broderick G., Fletcher M.A. (2012). Biomarkers for chronic fatigue. Brain Behav. Immun..

[30] Ruan Y., Xiang K.F., Zhang H.M., Qin Z., Sun Y., Wan J.J., Gu W., Liu X. (2022). Orosomucoid: A promising biomarker for the assessment of exercise-induced fatigue triggered by basic combat training. BMC Sports Sci. Med. Rehabil..

[31] Lei H., Sun Y., Luo Z., Yourek G., Gui H., Yang Y., Su D.F., Liu X. (2016). Fatigue-induced Orosomucoid 1 Actson C-C Chemokine Receptor Type 5 to Enhance Muscle Endurance. Sci. Rep..

[32] Kulkarni A.B., Reinke R., Feigelson P. (1985). Acute phase mediators and glucocorticoids elevate alpha 1-acid glycoprotein gene transcription. J. Biol. Chem..

[33] Calvin J., Pountain G.D., Hazleman B.L. (1995). Effect of short-term corticosteroid administration on the concentration of serum proteins. Ann. Clin. Biochem..

[34] Utrero-Rico A., Ruiz-Ruigómez M., Laguna-Goya R., Arrieta-Ortubay E., Chivite-Lacaba M., González-Cuadrado C., Lalueza A., Almendro-Vazquez P., Serrano A., Aguado J.M. (2021). A Short Corticosteroid Course Reduces Symptoms and Immunological Alterations Underlying Long-COVID. Biomedicines.

[35] Goel N., Goyal N., Nagaraja R., Kumar R. (2021). Systemic corticosteroids for management of ‘long-COVID’: An evaluation after 3 months of treatment. Monaldi Arch. Chest Dis..

[36] Myall K.J., Mukherjee B., Castanheira A.M., Lam J.L., Benedetti G., Mak S.M., Preston R., Thillai M., Dewar A., Molyneaux P.L. (2021). Persistent Post-COVID-19 Interstitial Lung Disease. An Observational Study of Corticosteroid Treatment. Ann. Am. Thorac. Soc..

[37] Kostorz-Nosal S., Jastrzębski D., Chyra M., Kubicki P., Zieliński M., Ziora D. (2021). A prolonged steroid therapy may be beneficial in some patients after the COVID-19 pneumonia. Eur. Clin. Respir. J..

[38] Kanczkowski W., Beuschlein F., Bornstein S.R. (2022). Is there a role for the adrenal glands in long COVID?. Nat. Rev. Endocrinol..

[39] Bonilla H., Quach T.C., Tiwari A., Bonilla A.E., Miglis M., Yang P., Eggert L., Sharifi H., Horomanski A., Subramanian A. (2022). Myalgic Encephalomyelitis/Chronic Fatigue Syndrome (ME/CFS) is common in post-acute sequelae of SARS-CoV-2 infection. medRxiv.

[40] Nijhof S.L., Rutten J.M., Uiterwaal C.S., Bleijenberg G., Kimpen J.L., Putte E.M. (2014). The role of hypocortisolism in chronic fatigue syndrome. Psychoneuroendocrinology.

[41] Harima A., Shimizu H., Takagi H. (1991). Analgesic effect of L-arginine in patients with persistent pain. Eur. Neuropsychopharmacol..

[42] Rondón L.J., Farges M.C., Davin N., Sion B., Privat A.M., Vasson M.P., Eschalier A., Courteix C. (2018). L-Arginine supplementation prevents allodynia and hyperalgesia in painful diabetic neuropathic rats by normalizing plasma nitric oxide concentration and increasing plasma agmatine concentration. Eur. J. Nutr..

[43] Severyanova L.A., Bobyntsev I.I., Kir’yanova N.A., Dolgintsev M.E. (2006). Effects of L-arginine on various types of pain sensitivity. Bull. Exp. Biol. Med..

[44] Böger R.H., Bode-Böger S.M., Thiele W., Creutzig A., Alexander K., Frölich J.C. (1998). Restoring vascular nitric oxide formation by L-arginine improves the symptoms of intermittent claudication in patients with peripheral arterial occlusive disease. J. Am. Coll. Cardiol..

[45] Rajapakse N.W., Mattson D.L. (2009). Role of L-arginine in nitric oxide production in health and hypertension. Clin. Exp. Pharmacol. Physiol..

[46] Izzo C., Visco V., Gambardella J., Ferruzzi G.J., Rispoli A., Rusciano M.R., Toni A.L., Virtuoso N., Carrizzo A., Di Pietro P. (2022). Cardiovascular implications of miRNAs in COVID-19. J. Pharmacol. Exp. Ther..

[47] Wittmann F., Prix N., Mayr S., Angele P., Wichmann M.W., van den Engel N.K., Hernandez-Richter T., Chaudry I.H., Jauch K.W., Angele M.K. (2005). L-arginine improves wound healing after trauma-hemorrhage by increasing collagen synthesis. J. Trauma.

[48] Schwarz D., Lipoldová M., Reinecke H., Sohrabi Y. (2022). Targeting inflammation with collagen. Clin. Transl. Med..

[49] Notarte K.I., Catahay J.A., Velasco J.V., Pastrana A., Ver A.T., Pangilinan F.C., Peligro P.J., Casimiro M., Guerrero J.J., Gellaco M.M.L. (2022). Impact of COVID-19 vaccination on the risk of developing long-COVID and on existing long-COVID symptoms: A systematic review. EClinicalMedicine.

[50] Notarte K.I., de Oliveira M.H.S., Peligro P.J., Velasco J.V., Macaranas I., Ver A.T., Pangilinan F.C., Pastrana A., Goldrich N., Kavteladze D. (2022). Age, Sex and Previous Comorbidities as Risk Factors Not Associated with SARS-CoV-2 Infection for Long COVID-19: A Systematic Review and Meta-Analysis. J. Clin. Med..

